# Radiomics Model Based on Non-Contrast CT Shows No Predictive Power for Complete Pathological Response in Locally Advanced Rectal Cancer

**DOI:** 10.3390/cancers11111680

**Published:** 2019-10-29

**Authors:** Gordian Hamerla, Hans-Jonas Meyer, Peter Hambsch, Ulrich Wolf, Thomas Kuhnt, Karl-Titus Hoffmann, Alexey Surov

**Affiliations:** 1 Department of Neuroradiology, University of Leipzig, 04103 Leipzig, Germany; Karl-Titus.Hoffmann@medizin.uni-leipzig.de; 2 Department of Diagnostic and Interventional Radiology, University of Leipzig, 04103 Leipzig, Germany; Hans-Jonas.Meyer@medizin.uni-leipzig.de (H.-J.M.); Alexey.Surov@uniklinik-ulm.de (A.S.); 3 Department of Radiooncology, University of Leipzig, 04103 Leipzig, Germany; Peter.Hambsch@medizin.uni-leipzig.de (P.H.); Ulrich.Wolf@medizin.uni-leipzig.de (U.W.); Thomas.Kuhnt@medizin.uni-leipzig.de (T.K.); 4 Department of Diagnostic and Interventional Radiology, Ulm University Medical Center, Albert-Einstein-Allee 23, 89081 Ulm, Germany

**Keywords:** rectal cancer, neoadjuvant chemoradiation, computed tomography, machine learning, algorithm, response prediction

## Abstract

(1) Background: About 15% of the patients undergoing neoadjuvant chemoradiation for locally advanced rectal cancer exhibit pathological complete response (pCR). The surgical approach is associated with major risks as well as a potential negative impact on quality of life and has been questioned in the past. Still, there is no evidence of a reliable clinical or radiological surrogate marker for pCR. This study aims to replicate previously reported response predictions on the basis of non-contrast CT scans on an independent patient cohort. (2) Methods: A total of 169 consecutive patients (126 males, 43 females) that underwent neoadjuvant chemoradiation and consecutive total mesorectal excision were included. The solid tumors were segmented on CT scans acquired on the same scanner for treatment planning. To quantify intratumoral 3D spatial heterogeneity, 1819 radiomics parameters were derived per case. Feature selection and algorithmic modeling were performed to classify pCR vs. non-pCR cases. A random forest model was trained on the dataset using 4-fold cross-validation. (3) Results: The model achieved an accuracy of 87%, higher than previously reported. Correction for the imbalanced distribution of pCR and non-PCR cases (13% and 87% respectively) was applied, yielding a balanced accuracy score of 0.5%. An additional experiment to classify a computer-generated random data sample using the same model led to comparable results. (4) Conclusions: There is no evidence of added value of a radiomics model based on on-contrast CT scans for prediction of pCR in rectal cancer. The imbalance of the target variable could be identified as a key issue, leading to a biased model and optimistic predictions.

## 1. Introduction

Consensus guidelines on the management of locally advanced rectal cancer have been evolving over the past years, changing direction from primary total mesorectal excision to the current standard of neoadjuvant chemoradiation [1,2]. The main goal of radiooncological treatment is to decrease the local recurrence rates. Response to neoadjuvant chemoradiation is a predictor of survival [3,4]. Furthermore, in 15% of patients the pathological assessment of the surgical specimen shows no detectable tumor cells, or pathological complete response (pCR) [4,5]. Compared to non-pCR, patients assessed as pCR exhibit a superior 5-year disease-free survival [4,6]. While the necessity for surgery has been questioned [7,8], the resection specimen still is required for determination of pCR. A number of risks are associated with surgical treatment, i.e., 2% risk of perioperative mortality, 11% risk of anastomotic leak, 5% risk of reoperation for complications, and risk of sexual and urinary dysfunction [7]. The clinical complete response (as determined by clinical, endoscopic and radiologic examination) failed to be a reliable surrogate in multiple studies [9,10,11,12].

Radiomics research is a fast-emerging field, enabling objectified analysis of routine medical imaging data [13]. Numerous scalar features can be derived from original and filtered radiological examinations, describing 3D spatial heterogeneity. Due to the potentially large amount of data to be processed, usually advanced statistical or algorithmic models are applied to filter redundant or uninformative features, and to find different phenotypes or prognostic patterns.

The development of imaging biomarkers for assessment of the therapy response in order to achieve an individualized tailored therapy is an issue of ongoing research [14]. In brief, radiomics can reflect tumor heterogeneity and thus, might be correlated to histopathology microstructure [15,16,17].

Recently, a machine learning model using a deep neural network and radiomics parameters derived from non-contrast CT scans for radiation therapy planning has been suggested as a means to predict pCR [18]. This is especially of interest because the non-contrast CT is routinely obtained for radiotherapy and is not diagnostically used. An opportunistic benefit of these images might be of crucial interest for the patients.

While MRI is the current standard in assessment and staging of rectal cancer, the purpose of the present study, was to analyze whether radiomics derived from non-contrast treatment planning CT can predict treatment response to radiotherapy in rectal cancers.

## 2. Results

### 2.1. Patients and Imaging

A total of 169 patients were included in this study, treated for a T2–T4 rectal carcinoma between February 2010 and February 2018 with neoadjuvant chemoradiation (mean total dose: 50.4 Gy, range 45.0–52.2 Gy) with infusional 5-fluorouracil (500–1000 mg/m² on days 1–5 and 29–33). All patients underwent MRI of the pelvis and a CT thorax and upper abdomen for staging, prior to therapy. Surgery was performed a mean 46.7 days after completion of radiotherapy. Mean follow-up was 34 months (range 2–95). Mean age was 56 years (41–90 years), there were 126 male and 43 female patients. Clinical information is summarized in Table 1. All scans were performed on the same CT scanner (Somatom Emotion, Siemens AG, Erlangen, Germany) using an unaltered non-contrast protocol (see Table 2). In this protocol, all parameters are fixed, including tube voltage and milliamperage.

### 2.2. Machine Learning Classification

**Figure 1:**
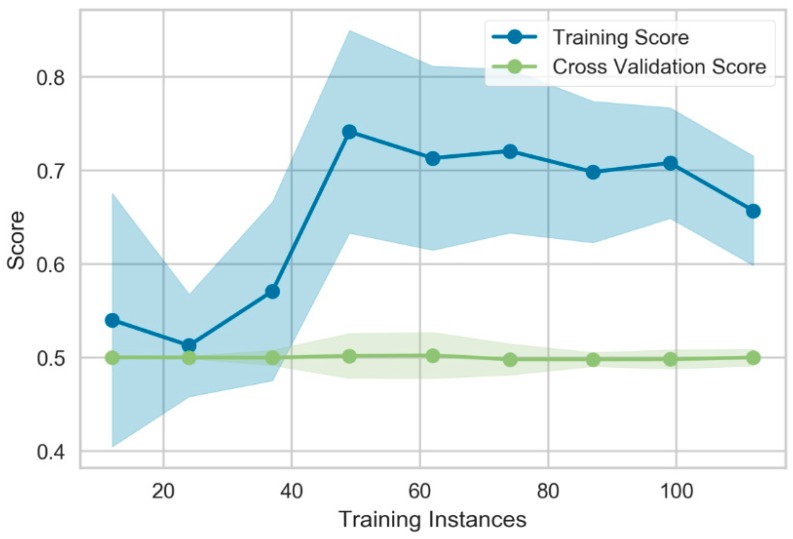
Learning curve for the random forest classifier for prediction of pCR after neoadjuvant chemoradiation in the rectal cancer cohort. Balanced accuracy as scoring method is depicted as a function of the used training instances. The cross-validation score remains on level with random results and does not converge, suggesting that the model would not benefit from a larger sample size.

Recursive feature elimination was carried out and the 63 most informative features were used to build the final model (see Table A1). Supervised machine learning (ML) was performed for the classification task pCR vs. non-pCR. The classifier was able to achieve a cross-validation test accuracy of 0.87 (SD: 0.01). Correction for the imbalanced group composition showed a balanced accuracy of 0.50 (SD: 0.01) and a Matthews correlation coefficient (MCC) of 0.0 (SD: 0.02). The learning curve produced in the evaluation process can help to assess the fitting results of the random forest model (Figure 1).

For internal validation, the whole process was repeated on an equally-sized set of computer-generated random values, keeping the unbalanced distribution of the target variables. On this set the random forest classifier was able to score an accuracy of 0.87 (SD: 0.01), a balanced accuracy of 0.5 (SD: 0) and an MCC of 0.0 (SD: 0.0).

## 3. Discussion

To our knowledge, this is the first study to replicate the use of a supervised machine learning classifier in predicting the complete pathological response (pCR) of rectal carcinomas to neoadjuvant radiotherapy, on the basis of treatment planning non-contrast CT scans as previously described by Bibault et al. [18]. These CT scans are routinely acquired for treatment planning only and are not used for diagnostic purposes. An opportunistic diagnostic benefit of these images using a radiomics approach might be of special interest for treatment evaluation.

As a result, we obtained an accuracy of 87% in our patient sample, which is outperforming the previously reported score (80% accuracy) [18]. Analysis of the model fitting process suggests that the measured prediction accuracy of 87% can resemble the performance of a random choice, most likely due to the imbalanced distribution of the target variable. The distribution of pCR in this series (pCR: non-pCR = 13%:87%) is similar to the distribution in the previously reported dataset (pCR:non-pCR = 23%:77%).

The difference of our primary results to those of the re-run of the analysis over a computer-generated random sample in an additional experiment is negligible. This bias issue arises when an algorithmic model is trained on an imbalanced dataset and it can lead to optimistic evaluations when the test set, too, is unevenly distributed [19]. In these cases, classification methods tend to be biased towards the majority class and the use of imbalance-correcting performance measures is recommended [20,21].

The learning curve depicts the balanced accuracy score for train and test datasets as a function of the cross-validation runs. The convergence of the curves for train and test scores towards a limit score can indicate a good fit of the model. On the current dataset however, the cross-validated test score remains on level with random results while the cross-validated training score converges towards 0.5, indicating a fail to predict the target variable using patterns in the available information. The lack of convergence of the test score during the learning process makes it unlikely that the model would benefit from a larger sample size.

Our data shows that there is no evidence for added value of random forest classification on radiomics data obtained from non-contrast planning scans for prediction of pCR to neoadjuvant chemoradiation. In conclusion, quantification of tumor density and shape, as derived from non-contrast CT scans, does not yield sufficient information on tissue pathology for this classification task. The use of a contrast agent-based CT imaging protocol on the other hand, facilitates modeling of clinical and histopathological parameters. In a 121 patient sample Vandendorpe et al. were able to predict clinical response to neoadjuvant chemoradiation with an AUC of 0.70 using contrast-enhanced CT and texture analysis [22]. Huang et al. proposed a model for discrimination of high-grade and low-grade adenocarcinomas in rectal cancer with an AUC of 0.72 using radiomics features derived from contrast-enhanced CT [23]. In conclusion, visually assessable size and shape features alone are not sufficient for modelling clinical and histopathological parameters in rectal cancer. More research is needed to assess the use of contrast-enhanced CT [24] and multiparametric MRI in comparison [25,26]. These advanced imaging modalities have been shown to be able to reflect histopathologic tissue properties like the Ki-67 proliferation index or receptor expression in the past [27]. For rectal cancer, a pooled analysis of two clinical phase II trials showed only fair interobserver agreement on complete response between the radiologist’s assessment of multiparametric MRI, and the pathologist’s assessment of the surgical specimen [9]. Yet, a radiomics model based on T2-weighted and diffusion-weighted MR imaging using a support vector machine classifier was able to achieve an AUC of 0.97 for pCR prediction [28]. Furthermore, using radiomics methodology MRI can be of help in predicting preoperative synchronous distant metastasis [29] and T stage [30].

A strength of this observation is the use of a single scanner with a fixed protocol, as radiomics parameters could be shown to lack reproducibility when acquisition parameters as the convolution kernel are altered [31]. Possible limitations of this work include the use of the random forest classifier. Its value for other classification tasks in radiomics research could be shown in other entities [32]. Bibault et al. used a densely-connected deep neural network as classifier, a more computationally intensive approach [18]. Rectal cancer can be a challenging target for segmentation due to circular and irregular growth patterns as well as invasive behavior. Thus, other segmentation strategies might yield different results. This sample consists of 169 cases, potentially limiting the classifier’s ability to learn representative patterns in the dataset and thus, ability to generalize. Repeated k-fold cross- validation was applied to cope with potential overfitting. Analysis of the fitting process revealed that the used model is unlikely to benefit from a larger sample size. A multivariate analysis was not performed.

## 4. Materials and Methods

### 4.1. Ethical Statement

This study was approved by the IRB and Ethics Committee of the University of Leipzig Medical Center (Date of decision: 28 August 2018). All experiments were carried out in accordance with relevant guidelines and regulations. The study used only pre-existing medical data; therefore patient consent was not required by the Ethics Committee.

### 4.2. Dataset Composition

**Figure 2:**
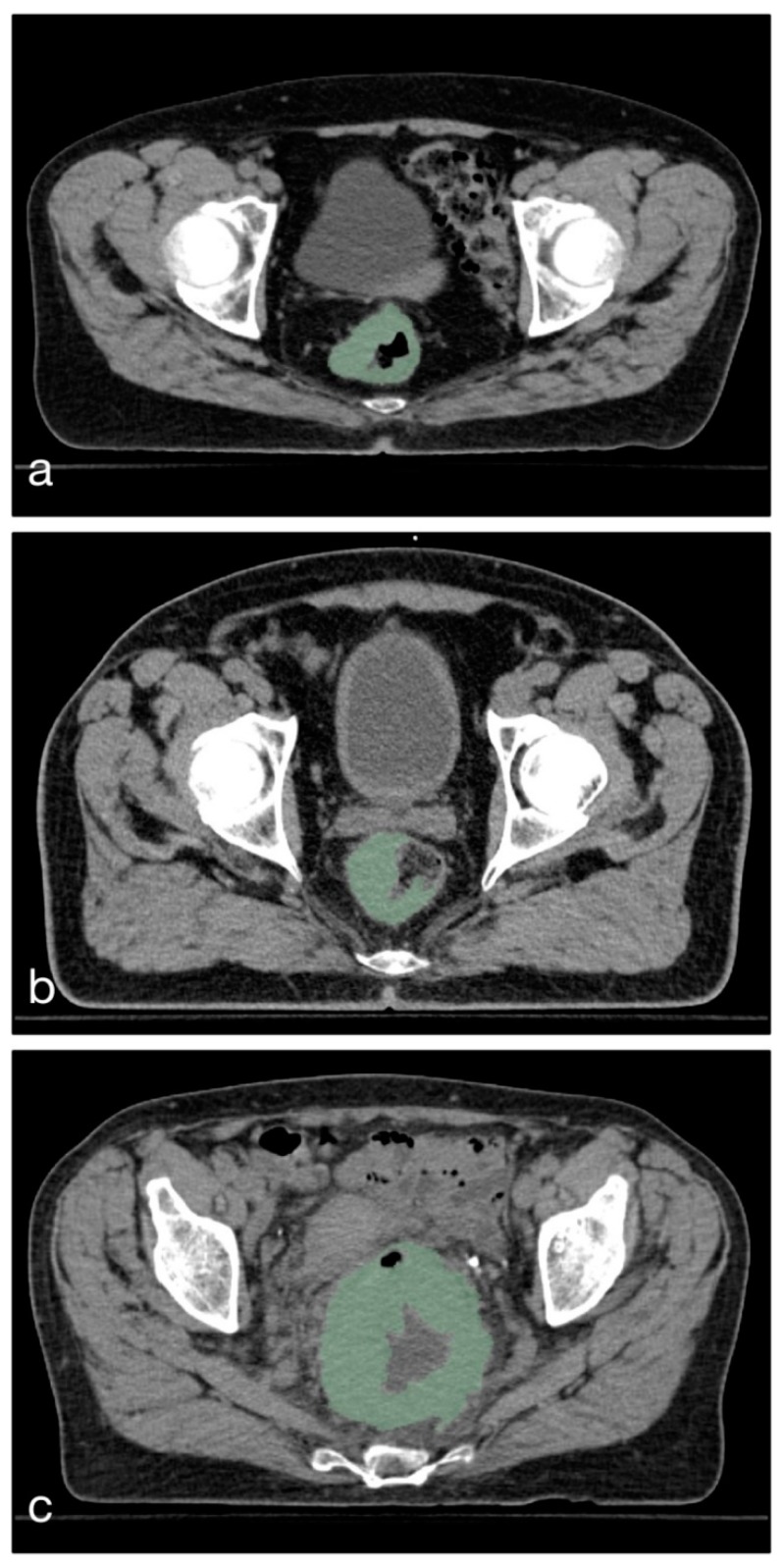
Three sample cases taken from the study population to illustrate the segmentation strategy. The segmented volume is marked in green color. Care was taken to only include tumor tissue (**a**) and exclude intraluminal contents (**b**), as well as surrounding tissue and gas inclusions (**c**).

For each case, the total tumor volume was manually delineated on the treatment planning CT scans acquired prior to radiotherapy slice by slice using 3D Slicer Software [33] and a digital pen tablet as input device (Wacom Europe GmbH, Düsseldorf, Germany). The segmentation was performed in consensus by a radiologist with 14 years of experience in abdominal imaging and a radiology resident with 3 years of experience. During segmentation, care was taken to include only voxels that resemble vital tumor tissue, avoiding intraluminal contents and adjacent tissue, as well as partial volume effects (see Figure 2).

The extraction of radiomics features was carried out using previously reported software [34], preserving the settings for possible future reproducibility. With regard to the highly standardized imaging, no normalization of Hounsfield units was performed. A full set of tumor image features was calculated for each case, including shape, first-order (histogram-derived) and texture features using the original as well as derived images (square, square root, logarithm, exponential, Laplacian of Gaussian, Wavelet reconstructions, gradient and logical binary pattern), resulting in 1819 features per scan.

Clinical information was gathered from the in-house digital documentation software (SAP AG, Walldorf, Germany).

### 4.3. Algorithmic Modeling

A random forest classifier (RF) was selected as the machine learning approach of choice [35]. The term ‘random forest’ refers to a set of tree-shaped structures that consist of a number of identically distributed random vectors. During training, within the decision tree each node is split using the best in a subset of the available features randomly chosen at that node [36]. This way, a set of the largest possible trees is grown and thus trained for the classification task at hand. The classification of a testing case by the trained tree set is determined by the majority vote. For the analysis of the radiomics dataset, an in-house developed Python-based software (Python 3.6.7) was used together with open-source statistics modules (numpy 1.17.2, scikit-learn 0.21.3) [37,38]. Parameters were left at default settings. The following steps of analysis were completed for the classification of pCR vs. non-PCR.

Recursive feature elimination was used for feature selection. In this approach, the model is fit repeatedly to the dataset and the number of features used to build the model are reduced by a defined step, leaving out the features of least importance, as determined by the classifier. The best feature subset is determined by the step with the maximum score.

Training and testing of the patient sample was performed by splitting the dataset into n = 4 equally-sized pieces, where n-1 pieces are used for training and the last piece as the testing dataset. The process is repeated n times and the average score is taken. This process is commonly referred to as n-fold cross-validation and can be used to achieve reliable results in small datasets. For scoring the classifier, the balanced accuracy of 100 repetitions with 4-fold cross-validation (CV) was used for scoring.

## 5. Conclusions

There is no evidence for added value of random forest classification on radiomics data obtained from non-contrast planning scans for prediction of pCR to neoadjuvant chemoradiation in locally advanced rectal cancer.

## Figures and Tables

**Table 1:** Clinical data of the patient cohort.

Items	Value	Range/Percent
Age (mean, range)	56 years	(41–90)
Sex		
Male	126	75%
Female	43	25%
T stage		
2	11	7%
3	136	80%
4	22	13%
N stage		
0	31	18%
1	89	53%
2	49	29%
Tumor volume (mean, range)	45.3 cm^3^	(3.3–483.6)
WHO Tumor Grading		
Grade 1	4	2%
Grade 2	128	76%
Grade 3	37	22%
Treatment		
Delivered Dose (mean, range)	50.4 Gy	(45–52.2)
Days to surgery (mean, range)	46.7 days	(9–124)
Follow-up (mean, range)	34 months	(2–95)
Outcome		
pCR (male/female)	22 (13/9)	13%
non-pCR (male/female)	147 (113/34)	87%

**Table 2:** CT protocol for treatment planning.

Item	Value
Scanner	Siemens Emotion (16 Slices)
Acquisition matrix	512 × 512
Voxel size	0.98 × 0.98 × 3 mm
Dose Modulation	None
Convolution Kernel	B40s
Contrast Agent	Non-contrast

## Appendix A

**Table A1:** List of features selected by recursive feature elimination for classification of pCR vs. non-pCR.

Features
wavelet-HHH_firstorder_Skewness
wavelet-HHH_glszm_SizeZoneNonUniformityNormalized
lbp-3D-k_glszm_ZoneEntropy
wavelet-HHH_glrlm_HighGrayLevelRunEmphasis
wavelet-HHH_glszm_ZoneVariance
wavelet-HHH_glrlm_LowGrayLevelRunEmphasis
wavelet-LHH_firstorder_RootMeanSquared
log-sigma-2-0-mm-3D_glrlm_LongRunHighGrayLevelEmphasis
wavelet-HHL_glszm_ZoneVariance
wavelet-HHL_glszm_LargeAreaEmphasis
wavelet-HHL_gldm_DependenceVariance
original_shape_Maximum2DDiameterSlice
wavelet-LHH_firstorder_Mean
log-sigma-3-0-mm-3D_gldm_SmallDependenceLowGrayLevelEmphasis
log-sigma-2-0-mm-3D_firstorder_Skewness
wavelet-HHL_glszm_LargeAreaHighGrayLevelEmphasis
log-sigma-3-0-mm-3D_gldm_LargeDependenceHighGrayLevelEmphasis
log-sigma-2-0-mm-3D_glrlm_RunEntropy
wavelet-LHH_firstorder_Uniformity
original_shape_MinorAxisLength
wavelet-LHL_glszm_SmallAreaEmphasis
wavelet-HLL_glszm_LargeAreaHighGrayLevelEmphasis
wavelet-HLH_firstorder_Kurtosis
log-sigma-3-0-mm-3D_gldm_LowGrayLevelEmphasis
wavelet-LHH_firstorder_Median
wavelet-LHH_glrlm_HighGrayLevelRunEmphasis
wavelet-LHL_glszm_ZoneVariance
gradient_firstorder_Minimum
log-sigma-2-0-mm-3D_glszm_ZoneEntropy
original_glszm_LargeAreaHighGrayLevelEmphasis
log-sigma-2-0-mm-3D_gldm_LargeDependenceHighGrayLevelEmphasis
log-sigma-2-0-mm-3D_glrlm_ShortRunLowGrayLevelEmphasis
wavelet-HHL_glszm_SizeZoneNonUniformityNormalized
log-sigma-2-0-mm-3D_glrlm_HighGrayLevelRunEmphasis
wavelet-LHH_glrlm_LowGrayLevelRunEmphasis
wavelet-HHH_glszm_SmallAreaHighGrayLevelEmphasis
log-sigma-2-0-mm-3D_glcm_Autocorrelation
log-sigma-3-0-mm-3D_glrlm_HighGrayLevelRunEmphasis
log-sigma-2-0-mm-3D_gldm_HighGrayLevelEmphasis
log-sigma-5-0-mm-3D_glcm_ClusterShade
log-sigma-2-0-mm-3D_glcm_JointAverage
log-sigma-5-0-mm-3D_glszm_ZoneEntropy
log-sigma-5-0-mm-3D_gldm_LowGrayLevelEmphasis
original_glszm_ZoneEntropy
log-sigma-5-0-mm-3D_glcm_Idmn
log-sigma-3-0-mm-3D_glcm_Idmn
wavelet-LHH_glszm_ZoneVariance
original_shape_LeastAxisLength
wavelet-LLL_glcm_Imc2
original_firstorder_10Percentile
wavelet-LHH_firstorder_Variance
wavelet-HHH_firstorder_Variance
wavelet-LLH_firstorder_Skewness
wavelet-LLH_glszm_SizeZoneNonUniformityNormalized
wavelet-LHH_gldm_GrayLevelVariance
original_glcm_Imc1
log-sigma-1-0-mm-3D_glrlm_RunLengthNonUniformity
log-sigma-5-0-mm-3D_glrlm_LowGrayLevelRunEmphasis
wavelet-LLL_glszm_LargeAreaHighGrayLevelEmphasis
wavelet-HHH_glszm_SmallAreaLowGrayLevelEmphasis
log-sigma-5-0-mm-3D_gldm_LargeDependenceLowGrayLevelEmphasis
wavelet-LHH_firstorder_MeanAbsoluteDeviation
wavelet-LHH_glszm_LargeAreaEmphasis
